# Human Milk Macronutrients and Bioactive Molecules and Development of Regional Fat Depots in Western Australian Infants during the First 12 Months of Lactation

**DOI:** 10.3390/life12040493

**Published:** 2022-03-28

**Authors:** Zoya Gridneva, Alethea Rea, Ching Tat Lai, Wan Jun Tie, Sambavi Kugananthan, Ashleigh H. Warden, Sharon L. Perrella, Kevin Murray, Donna T. Geddes

**Affiliations:** 1 School of Molecular Sciences, The University of Western Australia, Crawley, WA 6009, Australia; ching-tat.lai@uwa.edu.au (C.T.L.); ash.tie@uwa.edu.au (W.J.T.); he168801@health.wa.gov.au (S.K.); ashleigh.warden@uwa.edu.au (A.H.W.); sharon.perrella@uwa.edu.au (S.L.P.); donna.geddes@uwa.edu.au (D.T.G.); 2 Mathematics and Statistics, Murdoch University, Murdoch, WA 6150, Australia; alethea.rea@murdoch.edu.au; 3 School of Population and Global Health, The University of Western Australia, Crawley, WA 6009, Australia; kevin.murray@uwa.edu.au

**Keywords:** human milk, lactation, infants, regional body composition, intake, macronutrients, bioactive molecules, obesity, breastfeeding, fat depots

## Abstract

We investigated associations between intakes of human milk (HM) components (macronutrients and biologically active molecules) and regional fat depots development in healthy term infants (*n* = 20) across the first year of lactation. Infant limb (mid-arm and mid-thigh) lean and fat areas were assessed by ultrasound imaging at 2, 5, 9 and 12 months of age. Concentrations of HM total protein, whey protein, casein, adiponectin, leptin, lysozyme, lactoferrin, secretory IGA, total carbohydrates, lactose, HM oligosaccharides (total HMO, calculated) and infant 24-h milk intake were measured, and infant calculated daily intakes (CDI) of HM components were determined. This pilot study shows higher 24-h milk intake was associated with a larger mid-arm fat area (*p* = 0.024), higher breastfeeding frequency was associated with larger mid-arm (*p* = 0.008) and mid-thigh (*p* < 0.001) fat areas. Lysozyme (*p* = 0.001) and HMO CDI (*p* = 0.004) were time-dependently associated with the mid-arm fat area. Intakes of HM components and breastfeeding parameters may modulate infant limb fat depots development during the first year of age and potentially promote favorable developmental programming of infant body composition; however, further studies are needed to confirm these findings.

## 1. Introduction

Rapid fat mass (FM) gain in infancy is acknowledged as a risk factor for metabolic diseases in adulthood [1]. Breastfeeding is reportedly related to the development of infant body composition (BC) [2]; this relationship conceivably contributes to a lower risk of obesity and a lesser incidence of metabolic diseases [3]. Studies report that both subcutaneous-abdominal and visceral fat depots are independently and differentially regulated in infants [4,5] and that increased duration of exclusive breastfeeding is associated with increased subcutaneous but not visceral fat [4]. Furthermore, daily intakes of several human milk (HM) molecules were shown to have disparate associations with infant visceral and subcutaneous-abdominal adiposity [5], supporting the notion of protection against obesity [6]. This suggests that HM may ensure a beneficial (subcutaneous) adipose phenotype that is associated with a reduced risk of non-communicable diseases and obesity later in life [1,6]. However, the mechanisms of this protection are not currently demonstrated, further complicated by the longitudinal changes in HM composition [7] and the effects of maternal adiposity, diet and other environmental factors [8].

Measuring of infant BC would be prudent for assessment of infant growth in addition to anthropometrics and body mass index (BMI), as the latter is not recommended for clinical use in children under nine years of age being a poor predictor of relative body fat [9]. During the early months of infancy, BC measurements would provide important information for growth and nutritional assessment, enabling tailoring of early nutrition (energy and nutrient requirements of the infant), especially in high risk/preterm infants [10], yet they are difficult to obtain in this population [11]. The accuracy of whole BC measurements in infants is still being debated; there is also a clinical need for fast and reliable assessment of fat distribution and objective measurements of localised fat deposition [11].

Recently, ultrasound imaging has been validated for the assessment of the effect of macronutrients on preterm infants’ tissue accretion rates [12]. The study reported that ultrasound is sufficiently sensitive in the detection of the effects of daily macronutrient intakes, predominantly the intake of HM carbohydrates and protein energy ratio of intakes, on moderation of adipose-to-muscle tissue accumulation measured at four anatomical sites (mid-arm and mid-thigh lean and fat areas). Furthermore, the authors pointed out that macronutrient profiles and the timing of fortification have played a part in sculpting preterm BC. It is still not fully understood how breastfeeding and intakes of HM components influence the accretion of adipose and lean tissue in term healthy breastfed infants that feed on demand during and beyond the exclusive breastfeeding period.

The aim of this pilot study was to apply ultrasound imaging for measuring of regional BC of term infants to investigate relationships with daily intakes/concentrations of HM macronutrients and bioactive components during the first 12 months of breastfeeding on demand. Additionally, to establish relationships between infant limb adipose and lean tissue accretion and breastfeeding parameters, as well as maternal BC.

## 2. Methods

### 2.1. Participants and Design

We recruited 20 healthy (self-reported) English-speaking breastfeeding mothers with healthy infants from the community to visit our research laboratory at King Edward Memorial Hospital for Women (Subiaco, Perth, WA, Australia) during their infants’ first year of life (2, 5, 9 and 12 months after birth). Eligibility criteria were healthy singleton infants, birth gestation of ≥37 weeks, exclusively breastfed to 5 months and breastfed at 9 and 12 months. Exclusion criteria for infants were health issues potentially affecting growth and formula supplementation at any time points during the study; for mothers, low milk supply, gestational diabetes mellitus and smoking.

At the study visits, we collected HM samples and measured infant limb (mid-arm and mid-thigh) lean and fat areas as well as maternal anthropometry and BC. Participants arrived in the morning (09:30 a.m.–12:00 p.m.) to avoid circadian influence on the outcomes. Dyads have self-reported as healthy at the time of the study visit (no infectious illness, such as cold or flu, no indication of mastitis in mothers). Mothers measured infant 24-h milk intake (MI) and breastfeeding frequency (BFF, meals/24 h) at their homes by test-weighing their infants prior to and after every breastfeeding on up to 3 occasions: between 2 and 5 months, when MI is consistent [13,14], and within 2 weeks of both, 9 and 12 months.

### 2.2. Measurements of Infant Limb Fat and Lean Areas

**Figure 1:**
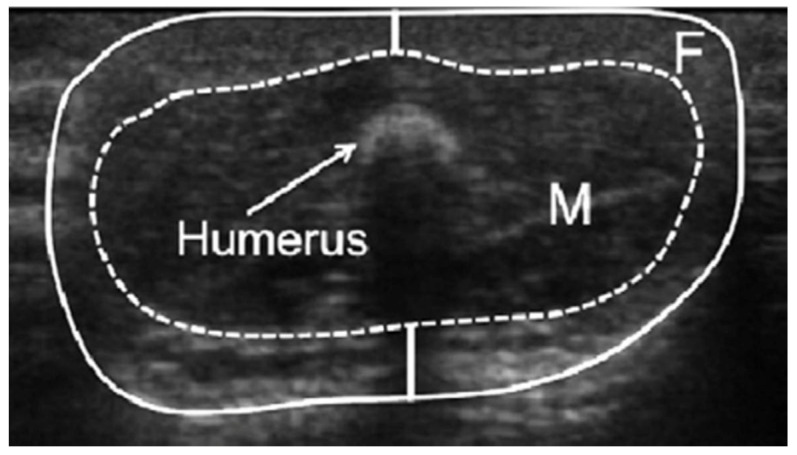
Ultrasound measurements of fat and lean areas of the infant’s arm. F, fat area; M, lean area (muscle and bone).

To assess infant regional adiposity, one experienced sonographer took single ultrasound measures with minimum compression using the Aplio XG (Toshiba, Tokyo, Japan) ultrasound machine with a high-resolution 14–8 MHz transducer (PLT-1204BX) and sterile water-based Aquasonic 100 US transmission gel (Parker Laboratories Inc., Fairfield, NJ, USA). Probe placement was on the anterior upper arm and thigh with the infant either supine or sitting on the mother’s/researcher’s lap. The infant mid-arm and mid-thigh lean and fat areas and circumferences were measured precisely from the images on the screen (Figure 1) according to previously validated protocols [12,15] using a universal desktop ruler. The sonographer previously displayed high intra-rater reliability [12].

### 2.3. Assessment of Maternal Body Composition

Maternal BC (fat-free mass (FFM), FM, %FM) was measured with bioelectrical impedance spectroscopy using the Impedimed SFB7 bioelectrical impedance analyser (ImpediMed, Brisbane, QLD, Australia) as reported formerly [16]. The within-participant coefficient of variation (CV) for maternal %FM was 0.21% [17]. The indices of maternal height–normalized BC were calculated: FFM index (FFMI) was calculated as FFM/height^2^; FM index (FMI) was calculated as FM/height^2^ [18].

### 2.4. Analysis of Human Milk Components

The methodology for analysis, together with concentrations and CDI of 11 HM components, were reported previously [17,19,20,21,22,23].

In short, pre-/post-feed samples were pooled (unless specified as not) and then defatted for measuring of all HM components [24] but the adiponectin [17] and leptin [19], which were measured in whole HM with ELISA. Casein and whey proteins were separated, as per Kunz and Lonnerdal [25] and Khan et al. [26]. Protein concentrations (casein, total and whey protein) were measured with the Bradford Protein assay [27]. Lactose was determined in pre- and post-feed samples using the enzymatic spectrophotometry and averaged for analysis [27]. For measurement of total carbohydrates, skim HM was deproteinized with trichloroacetic acid [28] and then dehydrated by sulfuric acid [29]. Lysozyme was measured by an adaptation of Selsted and Martinez methods [30,31], lactoferrin and sIgA were measured with ELISA [31,32]. Standard assays were adapted for and carried out using a JANUS workstation (PerkinElmer, Inc., Waltham, MA, USA), measurements were performed on EnSpire (PerkinElmer, Inc., Waltham, MA, USA). We conducted all measures in duplicate and averaged results for statistical analysis. The total HM oligosaccharide (HMO) concentration was calculated by deducting lactose concentration from total carbohydrates concentration.

We used 24-h MI values from the 24-h test-weighing [13] and component concentrations measured in HM samples collected at the research laboratory visit to estimate calculated daily intakes (CDI), which were considered representative of a typical daily intake at the corresponding time point.

### 2.5. Statistical Analyses

The study design as well as power calculation and statistical analyses used for this cohort have been reported previously [16,20,21,22]. Briefly, during this longitudinal pilot study, we measured infants at four time points (2 and/or 5, 9 and 12 months). As there is no closed-form expression suitable for the calculation of sample sizes for longitudinal study [33], we calculated an approximate sample size as if this was a cross-sectional study with equal numbers at each time point [34]. Allowing four predictors (3 for age comparisons), α = 0.05 and 14 participants (56 sample points) gave the study power of 0.80 to detect an effect size of 0.15, with the consideration that longitudinal study design is more powerful. We introduced recruitment of participants at the 5-month point, as many mothers would not commit to a study that required breastfeeding to 12 months when approached at 2 months (*n* = 8). We increased participant number to 20 to ensure the predicted power; this also addressed issues relating to missed visits. Missing data were dealt with using available case analysis.

Relationships between variables were analysed using linear mixed-effects models. Fitted models included a response (infant limb fat and lean area measurements) and an explanatory variable: (a) HM component concentrations; (b) HM components CDIs; (c) breastfeeding parameters (24-h MI, BFF); and (d) maternal BC measures. The fixed effects were infant age (as a categorical variable), the explanatory variable of interest and interaction with the explanatory variable of interest and age as well as a random effect for each participant. If the *p*-value associated with the interaction was below 0.05, the results were reported for the full model (fixed effects for infant age, the explanatory variable of interest and the interaction); otherwise, results are reported for the model fitted without interaction (fixed effects for infant age and the explanatory variable of interest). Data were checked for statistical outliers and where the identified outlier had a significant impact the results are reported for both.

Systematic differences between measured parameters at 2, 5, 9 and 12 months were analysed using linear mixed model (infant age as a fixed factor, participant as a random factor). We used general linear hypothesis tests (Tukey’s all-pair comparisons) to analyse the differences between the time points.

A false discovery rate (FDR) adjustment was applied to the subgroupings of results to the interaction *p*-value if it was less than 0.05 or to the main effect *p*-value [35]. The adjusted significance levels are reported in the Tables and set at <0.05 otherwise. We reported results as mean ± standard deviation (SD) and range and as parameters estimates ± standard error (SE). We used R 4.0.2 to perform the statistical analysis and visuals.

## 3. Results

### 3.1. Participants

**Figure A1:**
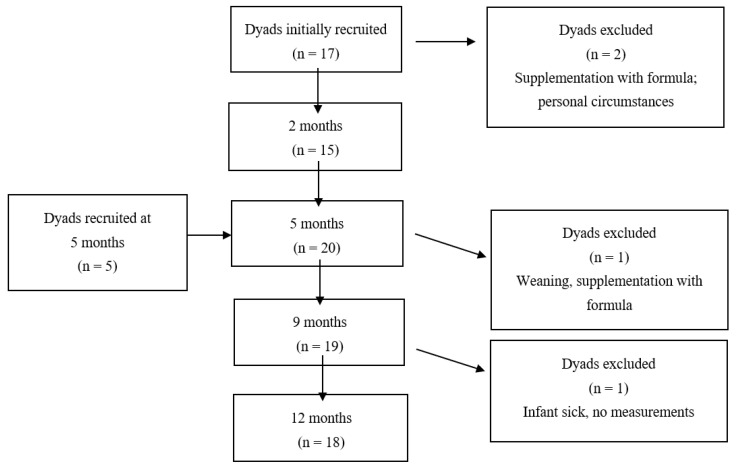
Flowchart of study participants.

Participants’ demographics, anthropometrics and BC measured at the study sessions, 24-h MI and BFF, and the sample sizes at all time points are shown in Table 1 and Figure A1 and have been reported previously together with HM components concentrations and CDIs and attrition and missing data [16,20,21,22,23]. Briefly, mothers were mainly of European ancestry and higher social-economic status. Maternal age at the commencement of the study was 33.3 ± 4.7 (24–44) years, height was 167.4 ± 7.4 (150–181) cm and parity was 2.3 ± 0.9 (1–4). Infant birth weight was 3.486 ± 0.498 (2.660–4.455) kg and gestational age was 39.4 ± 1.32 (37.6–43) weeks. Missing data occurred due to some participants not attending all sessions and the difficulties with conducting 24-h MI measurements at later stages of lactation and included: (a) measurements of mid-arm and mid-thigh lean and fat areas (*n* = 18 from the 80 anticipated); (b) CDI of casein, whey and total protein, lactose, adiponectin, leptin, lactoferrin and sIgA (*n* = 27), CDI of total carbohydrates and HMOs (*n* = 28), and lysozyme CDI (*n* = 30 from the 60 anticipated).

### 3.2. Infant Limb Fat and Lean Areas across the First 12 Months of Age

The longitudinal changes in maternal and infant whole BC, 24-h MI and BFF as well as concentrations and CDI of HM components have been published earlier [16,20,21,22,23]. Infant limb measures across the 12 months of life are presented in Table 1. In this cohort, lean regional areas increased significantly as age increased, mid-arm fat areas initially increased and then plateaued and decreased, whilst mid-thigh fat areas increased significantly from 2 to 5 months with no further significant changes (Table 2).

### 3.3. Human Milk Components and Infant Limb Fat and Lean Areas

**Figure 2:**
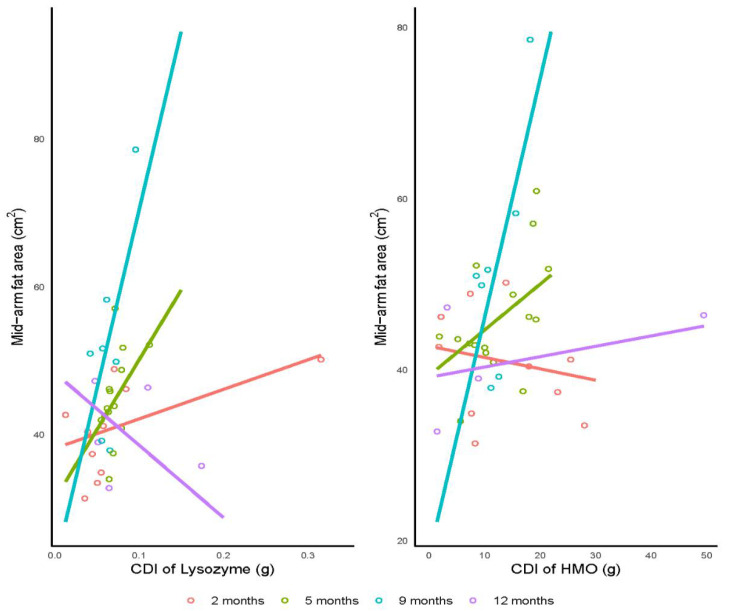
Significant associations between infant mid-arm fat area and calculated daily intakes (CDI) of lysozyme (overall *p* = 0.001, prior to the outlier removal) and human milk oligosaccharides (HMO; overall *p* = 0.004). Lines represent linear regression and grouped by the infant age.

This pilot study suggests that lysozyme CDI may be positively associated with infant mid-arm fat area at 2, 5 and 9 months and negatively at 12 months (overall *p* = 0.001). However, when a biological outlier is removed, the association does not stand (*p* = 0.24). HMO CDI was negatively associated with the mid-arm fat area at 2 months and positively at 5, 9 and 12 months (overall *p* = 0.004), with no further associations for either CDI or concentrations of HM components following the FDR adjustment (Table 3, Figure 2).

### 3.4. Breastfeeding Parameters and Infant Limb Fat and Lean Areas

**Figure 3:**
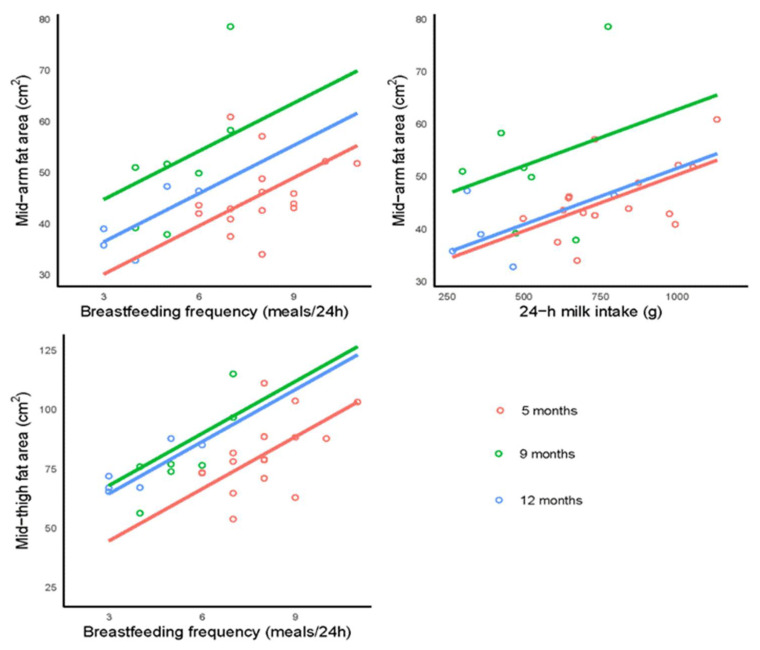
Significant associations between infant mid-arm fat area and breastfeeding frequency (*p* = 0.008) and 24 h milk intake (*p* = 0.024), and between infant mid-thigh fat area and breastfeeding frequency (*p* < 0.001). Lines represent linear regression and grouped by the infant age.

This pilot study suggests that higher infant 24-h MI may be associated with larger infant mid-arm fat area (*p* = 0.024) whilst higher BFF was associated with larger both, mid-arm (*p* = 0.008) and mid-thigh (*p* < 0.001) fat areas (Table 4, Figure 3). No significant associations between breastfeeding parameters and infant lean limb areas were found following the FDR adjustment.

### 3.5. Maternal Body Composition and Infant Limb Fat and Lean Areas

Maternal BC and anthropometry were not associated with infant limb lean and fat areas (data not presented).

## 4. Discussion

**Figure 4:**
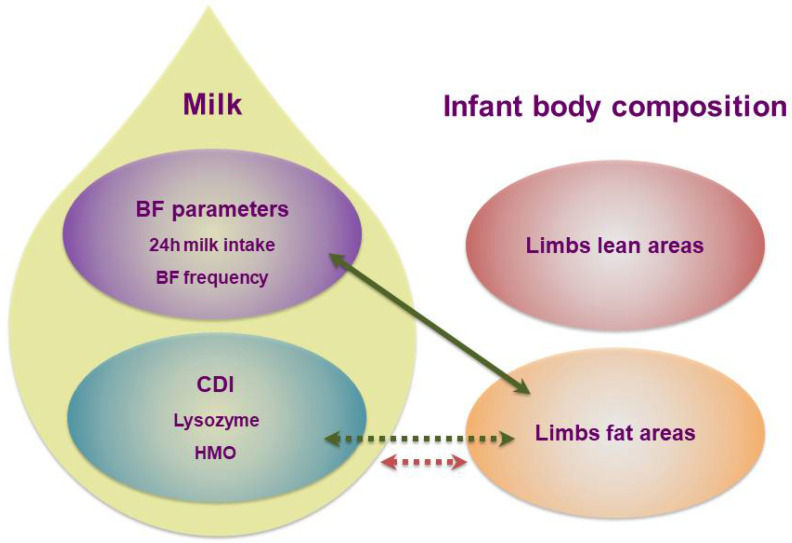
Potential pathways of lactocrine programming of the infant regional adiposity across the first year of lactation. Associations between predictors and infant regional adiposity are indicated by the arrows (green-positive; red-negative); dotted arrows indicate time-dependent associations. BF-breastfeeding; CDI–calculated daily intakes; HMO–human milk oligosaccharides.

This pilot longitudinal study examines some of the potential mechanisms by which breastfeeding and HM components may reduce the risk of obesity later in life. Primary we have concentrated on the effect of the doses (CDI) of a multitude of HM macronutrients and bioactive molecules on term infant regional adiposity during the first 12 months of breastfeeding and found suggestions that regional adipose tissue depots in arm and thigh are disparately regulated during infancy. Foremost HM composition and component intakes and infant MI and BFF are potentially linked to the development of infant regional subcutaneous adiposity (Figure 4). With CDIs of HM components relating to subcutaneous fat depots, our findings suggest that breastfeeding and HM may be protective against obesity; however, further work is needed in a larger number of dyads to confirm these findings.

Humans are the fattest species at birth, born with approximately 15% total body fat [36], which peaks at around 25% between 6–9 months of age [37] before gradually declining. Most of the energy derived from infant adipose tissue is used as an energetic buffer to support infant brain function between the feeds [38] and to aid with marginal nutrition during illness and the introduction of solids. Furthermore, the breakdown products of fat oxidation and ketones not only provide an alternative to glucose to fuel the brain, they are also the building blocks (carbon) for developing brain cells. This contributes to mild infant ketonemia (permanently elevated ketone circulation levels regardless of feeding status) from as early as mid-gestation [39]. Additional to mother’s milk, infant body fat also provides DHA for the membranes of the developing brain [38].

The evolutionary advantages of having higher infant fat accretion at birth are further supported by breastfeeding. The fat accretion in exclusively breastfed infants is reportedly higher than in formula-fed [2,40] and partially breastfed infants [41]. Furthermore, at 3 and 6 months of age, exclusive breastfeeding duration is positively related to infant subcutaneous–abdominal fat and %FM [42]. This indicates that fat accumulation during the period of active growth during infancy may be crucial for the programming of infant BC and health later in life. Whilst children with obesity are more likely to become adults with obesity and obesity-related non-communicable diseases [43,44] and increased BMI during infancy is positively associated with adult BMI, %FM and FFM [45], there are no data on whether infant regional body composition persists into adulthood. In adults, the location of fat accretion may predetermine the metabolic risk, with higher fat accretion in the abdominal region associating with higher risk [46] and unfavourable glucose and lipid levels [47]. Paradoxically, an increase of fat in the legs is associated with a lower risk of cardiovascular disease and lower cardiometabolic risk factors [48,49] and with more favourable levels of glucose [47,50] and lipids [47]. Fully breastfed infants with high adiposity are sometimes considered to be ‘overfed’ and at risk of later metabolic disease. However, published reports of breastfed infants with high BMI-for-age and adiposity showed typical 24-h MI and macronutrient composition in the first 6 months and subsequent catch-down growth [51,52]. This concurs with findings from this study that CDI of specific HM components influence BC in breastfed infants.

Adding to these reports, the suggestions of this study are the prospects of time-dependent and predominantly positive associations between CDI of lysozyme (prior to the outlier removal) and total HMOs and infant limb fat areas. Our previous investigations in the same cohort showed the possibility of several time-dependent differential associations of CDI of HM macronutrients and bioactive molecules as well as 24-h MI and BFF with infant adiposity (FM), supporting the current findings. Higher CDI of lysozyme was previously suggested to be associated with increased infant FM and FMI [23]; however, similar associations of total HMO CDI were rendered non-significant after the FDR adjustment [22]. Lysozyme [53,54,55] and HMOs [56,57,58,59] are found in HM in high concentrations and both are biologically active and anti-pathogen, modulating the infant gut microbiome and potentially affecting BC development. Studies of either HM lysozyme [60] or oral supplementation with bovine lysozyme [61] have shown increased weight gains in preterm infants, similar to the findings of this study. A recent study also reported infant intakes of individual and total acidic HMOs were positively associated with infant FM and weight-for-age and weight-for-length z-scores between 2 and 6 months after birth [62].

We also did not observe any associations with concentrations of lysozyme or total HMOs, nor did we find any strong associations between concentrations of other HM components and lean and fat areas of the limbs. However, prior to FDR adjustment, multiple time-dependent relationships between concentrations of several macronutrients and bioactive components and both lean and fat areas were observed, which were overall predominantly negative (Table 3). Additionally, HM leptin concentration, which was measured in whole milk, had a negative relationship with mid-thigh lean areas, which is in line with the previously reported (also prior to FDR) relationship of whole milk leptin with infant FFM [20]. Adiponectin concentration related negatively to mid-thigh fat areas, which is also similar to the studies that reported negative relationships between HM adiponectin and subcutaneous-abdominal depth [5] and weight-for-age and weight-for-length z-scores [63,64] as well as zBMI score [64].

Additionally, prior to FDR, HM protein concentrations showed the possibility of predominantly negative associations with limb fat areas (total protein, casein) and lean areas (whey protein). Recent studies that explored the relationships of HM macronutrients with regional adiposity reported a negative association between total protein concentration and visceral fat thickness [65] and preperitoneal fat area (prior to FDR) [5]. The relationships of two other immunomodulatory proteins, lactoferrin and sIgA, were more complicated, with both negatively associating with limb lean areas during exclusive breastfeeding and positively afterwards. sIgA, however, positively related to limb fat areas during exclusive breastfeeding, then the relationship became negative. Lactoferrin and sIgA previously were reported to relate differentially and time-dependently to infant visceral depth (a proxy measure for visceral fat) [5]. However, in the current analysis, none of these described associations have been supported by the associations at the CDI level, highlighting the importance of measuring MI in future studies.

Another potential finding is that infants that consumed more milk and fed more frequently had higher limb fat accretion. MI is a prime determining factor of growth during infancy [27,66]; within the same cohort we have reported previously that higher BFF is associated positively with 24-h MI, and that both, 24-h MI and BFF related positively to infant adiposity and negatively to lean mass [16]. In a larger longitudinal cohort of exclusively breastfed infants, we established that smaller, shorter infants with lower %FM, yet not the younger ones, had a higher BFF [67]. These and similar findings hint that infant BC drives the relationship of MI with infant growth [68] and further support feeding on demand.

In this analysis, we have not reported any potential relationships between maternal whole BC and infant regional BC, which is contradictory to studies of infant whole BC. Maternal adiposity previously has been reported to relate negatively to infant FFM [16] and subcutaneous-abdominal fat areas [5], as well as to infant adiposity, lean mass and z-scores [69]. Our results suggest that regional BC, whilst contributing to infant whole BC, may be regulated differentially and some regional adiposity, such as limb fat depots, may not be affected by maternal adiposity, thus increased maternal adiposity may not automatically reduce some of the protective effects of breastfeeding against obesity.

Our findings highlight the importance of accurate measurements of regional BC when assessing growth and potential health risks. This ultrasound method of measuring infant regional adiposity could be used to explore relationships between infant early nutrition and development of BC and potentially, as a tool in the clinical setting and nutritional interventions.

This pilot study concentrated on infants that breastfed on demand during the first year of life; it is pensive of normal lactation and advancement of infant regional BC. The strengths of this investigation are the longitudinal measures of participants and sampling of milk, as well as of actual 24 MI and CDIs of HM components, and the broad diversity in maternal BC. The study limitations are the small participant numbers linked to the numerous measurement time points and the particularly limited number of 24-h MI measures after the introduction of solids and an estimation of the total HMO concentration, notwithstanding the technical difficulties with accounting for all individual HMOs. We were unable to collect data on infant dietary intake from solids after 5 months of age, or on maternal diet, which might influence infant BC [70]; however, any compositional changes in HM, particularly in HM metabolic hormones, may be driven by maternal adiposity rather than diet [8,71,72]. Our sample consisted of breastfed singletons born at term to Western Australian urban mothers with mainly European ancestry and of higher social-economic status; thus, our findings are likely not representative of more diverse populations.

## 5. Conclusions

CDI of HM components and breastfeeding parameters may modulate the development of infant limb fat depots during the first 12 months of breastfeeding and potentially contribute to beneficial developmental programming of infant regional BC. Studies of larger and more diverse longitudinal cohorts are needed to confirm these findings.

## Figures and Tables

**Table 1:** Participant anthropometric, limb lean and fat areas and breastfeeding parameters.

Characteristics	2 Months	5 Months	9 Months	12 Months
	Mean ± SD (Min–Max)	Mean ± SD (Min–Max)	Mean ± SD (Min–Max)	Mean ± SD (Min–Max)
Mothers	(*n* = 14)	(*n* = 20)	(*n* = 18)	(*n* = 18)
Weight (kg)	78.8 ± 19.3 ^a^	70.1 ± 17.8	63.0 ± 10.0	64.2 ± 17.3
	(57.5–116.2)	(53.7–115.3)	(50.4–121.9)	(51.4–121.9)
BMI (kg/m^2^)	27.2 ± 5.5	24.8 ± 5.0	22.7 ± 3.9	23.9 ± 5.9
	(20.4–35.5)	(19.0–35.2)	(17.9–37.2)	(18.2–37.2)
Infants	(*n* = 15)	(*n* = 20)	(*n* = 19)	(*n* = 18)
Sex (M/F)	9 M/6 F	10 M/10 F	10 M/9 F	9 M/9 F
Age (months)	2.04 ± 0.14	5.16 ± 0.22	9.22 ± 0.27	12.26 ± 0.28
	(1.87–2.33)	(4.77–5.47)	(8.83–9.77)	(11.63–12.67)
Length (cm)	58.1 ± 1.9	64.8 ± 2.3	71.7 ± 1.9	73.6 ± 3.2
	(54.2–60.0)	(60.5–69.5)	(66.0–74.0)	(69.0–78.5)
Weight (kg)	5.63 ± 0.66	7.43 ± 1.13	8.84 ± 0.98	9.65 ± 0.62
	(4.42–7.40)	(5.81–9.51)	(6.68–10.10)	(7.17–11.09)
BMI (kg/m^2^)	16.6 ± 1.2	17.6 ± 1.9	17.7 ± 1.7	17.8 ± 0.9
	(14.5–18.1)	(14.9–20.4)	(14.2–20.2)	(13.7–19.2)
Infant limbs measurements	(*n* = 13)	(*n* = 19)	(*n* = 18)	(*n* = 12)
Mid-arm lean area (cm^2^)	106.6 ± 11.8	116.6 ± 10.4	125.2 ± 12.7	127.3 ± 6.9
	(82.4–120.8)	(98.1–134.3)	(106.4–156.4)	(118.5–142.5)
Mid-arm fat area (cm^2^)	41.6 ± 6.6	47.2 ± 7.5	49.6 ± 9.3	40.5 ± 6.7
	(31.4–53.0)	(34.0–62.3)	(37.9–78.6)	(31.4–50.0)
Mid-thigh lean area (cm^2^)	148.3 ± 21.6	179.2 ± 22.5	197.0 ± 24.9	195.3 ± 29.0
	(110.5–184.6)	(138.2–214.2)	(170.8–270.8)	(117.9–237.7)
Mid-thigh fat area (cm^2^)	61.8 ± 12.5	81.0 ± 16.5	76.9 ± 24.4	71.2 ± 15.5
	(41.3–85.5)	(52.4–112.2)	(22.0–121.2)	(41.3–91.6)
Breastfeeding parameters				
24-h MI (g)	(*n* = 17) ^b^		(*n* = 8)	(*n* = 8)
	819 ± 205		478 ± 154	451 ± 216
	(498–1185)		(300–775)	(255–795)
BFF (meals/24 h)	(*n* = 17) ^b^		(*n* = 8)	(*n* = 9)
	8.1 ± 1.4		5.4 ± 1.3	4.4 ± 2.1
	(6–11)		(4–7)	(2–8)

BFF, breastfeeding frequency; BMI, body mass index; M/F, male/female; MI, milk intake; MP, milk production; n/a not applicable. ^a^ Data are mean ± SD and ranges. ^b^ 24-h MI and BFF (meals/24-h) were measured between 2–5 months and within 2 weeks of 9 and 12 months.

**Table 2:** Significant differences by lactation duration within infant regional lean and fat areas ^a^.

	Months after Birth						
Infant Characteristic	Between 5 and 2 Months	Between 9 and 2 Months	Between 12 and 2 Months	Between 9 and 5 Months	Between 12 and 5 Months	Between 12 and 9	Overall *p*-Value
	(*n* = 13)	(*n* = 13)	(*n* = 12)	(*n* = 18)	(*n* = 12)	(*n* = 12)	(*n* = 18)
Mid-arm lean area (cm^2^)	10.93 ± 3.17 ^b^ (**0.003**) ^c^	19.19 ± 3.22 (**<0.001**)	22.73 ± 3.75 (**<0.001**)	8.26 ± 2.86 (**0.020**)	11.80 ± 3.40 (**0.003**)	3.54 ± 3.42 (0.73)	<0.001
Mid-arm fat area (cm^2^)	5.76 ± 2.45 (0.087)	8.00 ± 2.49 (**0.007**)	−2.09 ± 2.88 (0.89)	2.24 ± 2.22 (0.74)	−7.84 ± 2.62 (**0.015**)	−10.08 ± 2.64 (**<0.001**)	<0.001
Mid-thigh lean area (cm^2^)	30.34 ± 8.04 (**0.001**)	48.06 ± 8.15 (**<0.001**)	46.95 ± 8.93 (**<0.001**)	17.72 ± 7.29 (0.071)	16.61 ± 8.08 (0.17)	−1.12 ± 8.15 (1.00)	<0.001
Mid-thigh fat area (cm^2^)	16.96 ± 5.88 (**0.020**)	12.71 ± 5.97 (0.14)	6.48 ± 6.57 (0.76)	−4.25 ± 5.33 (0.86)	−10.48 ± 5.92 (0.29)	−6.23 ± 5.97 (0.72)	0.035

^a^ Systematic differences in the measured parameters between different time points were determined with general linear hypothesis test (Tukey’s all pair comparisons). ^b^ Data are parameter estimate and standard error of estimate. ^c^ *p*-value, bold font indicates significant difference (*p* < 0.05) between two time points.

**Table 3:** Associations between infant fat and lean limb areas, and daily intakes and concentrations of human milk components.

Predictor	2 Months		5 Months		9 Months		12 Months		*p*-Values		
	Intercept (SE)	Slope (SE)	Intercept (SE)	Slope (SE)	Intercept (SE)	Slope (SE)	Intercept (SE)	Slope (SE)	Predictor	Infant Age (Months)	Interaction
Daily intakes of milk components											
	(*n* = 13)		(*n* = 17)		(*n* = 7)		(*n* = 5)		(*n* = 17)		
Mid-arm lean area (cm^2^), significant at <0.005 ^d^											
Leptin (ng/day)	105 (10.3)	0.01 (0.02) ^a^	133 (6.82)	−0.05 (0.02)	106 (14.3)	0.08 (0.05)	128 (9.58)	−0.004 (0.04)	0.32	0.019	0.046 ^b^
Mid-arm fat area (cm^2^), significant at <0.009											
Lysozyme (g/day)	38.2 (2.79)	40 (23.2)	31.2 (9.09)	189 (125)	21.9 (9.95)	484 (151)	48.4 (5.99)	−98.1 (58.4)	0.064	<0.001	**0.001**
HMO (g/day)	42.8 (4.05)	−0.13 (0.25)	39.3 (4.16)	0.54 (0.30)	18.1 (10.7)	2.79 (0.84)	39.1 (4.58)	0.12 (0.18)	0.28	0.002	**0.004**
Mid-thigh lean area (cm^2^), significant at <0.005											
Leptin (ng/day)	179 (13.7)	−0.06 (0.03)	202 (10.5)	−0.06 (0.03)	215 (10.1)	−0.06 (0.03)	215 (9.63)	−0.06 (0.03)	0.025 ^c^	0.001	0.16
Concentrations of milk components											
	(*n* = 13)		(*n* = 19)		(*n* = 18)		(*n* = 9)		(*n* = 19)		
Mid-arm lean area (cm^2^), significant at <0.005											
Lactoferrin (g/L)	119 (6.27)	−26.4 (10.9)	122 (6.9)	−12.1 (15.5)	115 (6.21)	15.7 (9.51)	119 (12.1)	13.8 (17.1)	0.73	<0.001	0.029
Lactose (g/L)	185 (32)	−1.18 (0.47)	88.9 (33.9)	0.42 (0.52)	85.7 (30.1)	0.60 (0.46)	151 (42.1)	−0.33 (0.63)	0.73	<0.001	0.040
sIgA (g/L)	126 (7.93)	−36.4 (13.5)	105 (6.82)	22.6 (12.5)	118 (7.3)	11 (11.2)	120 (11.8)	11.2 (16.3)	0.89	<0.001	0.007
Mid-arm fat area (cm^2^), significant at <0.005											
sIgA (g/L)	34.7 (5.99)	12.9 (10.2)	46.6 (5.14)	1.91 (9.47)	63.8 (5.49)	−22.7 (8.46)	43.4 (8.87)	−7.11 (12.3)	0.38	<0.001	0.041
Mid-thigh lean area (cm^2^), significant at <0.005											
Whey protein (g/L)	206 (28.1)	−8.78 (4.14)	254 (32.1)	−13.6 (5.78)	164 (20.8)	5.41 (3.3)	230 (27.7)	−4.35 (3.86)	0.15	<0.001	0.008
Lactoferrin (g/L)	167 (13.7)	−36.2 (24.1)	211 (14.9)	−75.5 (33.7)	180 (13.4)	28 (20.7)	207 (21.2)	−10.8 (31.7)	0.35	<0.001	0.040
Mid-thigh fat area (cm^2^), significant at <0.005											
Adiponectin (ng/mL)	78.8 (10.8)	−1.31 (0.92)	99.4 (10.7)	−1.84 (1.04)	143 (20.6)	−7.72 (2.31)	71.9 (15.4)	−0.144 (1.34)	0.039	0.023	0.028
Total protein (g/L)	159 (34.3)	−8.65 (3.14)	975 (14.5)	0.58 (1.2)	111 (24.1)	−3.14 (2.28)	72.5 (21.6)	−0.352 (1.79)	0.32	0.009	0.030
Casein (g/L)	54.6 (23.2)	9.61 (18.5)	84.4 (10.5)	−1.45 (5.71)	116 (13.8)	−32.2 (10.9)	88.3 (19.2)	−13.7 (13)	0.19	0.004	0.039

^a^ Data are parameter estimate ± SE (standard error of estimate); effects of predictors taken from linear mixed-effects models that accounted for infant age and interaction between infant age and predictor with a random effect for the participant; if the *p*-value for interaction is not <0.05 parameter estimates are taken from a model with no interaction. ^b,c^ Results are presented only for interactions or predictors with raw *p*-values < 0.05. ^d^ Significance for the tested combinations after the FDR adjustment (significant *p*-values are indicated by the bold text). HMO, human milk oligosaccharides; sIgA, secretory immunoglobulin A.

**Table 4:** Associations between breastfeeding parameters and infant fat and lean limb areas.

Predictor	2–5 Months		9 Months		12 Months		*p*-Values		
	Intercept (SE)	Slope (SE)	Intercept (SE)	Slope (SE)	Intercept (SE)	Slope (SE)	Predictor	Infant Age (Months)	Interaction
	(*n* = 14)		(*n* = 7)		(*n* = 5)		(*n* = 14)		
Mid-arm fat area (cm^2^), significant at <0.05 ^c^									
BFF (meals/24 h) ^d^	20.7 (9.88) ^a^	3.15 (1.21)	35.3 (7.23)	3.15 (1.21)	27 (6.22)	3.15 (1.21)	**0.008** ^b^	0.002	0.065
24 h MI (g) ^d^	28.9 (7.65)	0.021 (0.009)	41.4 (5.64)	0.021 (0.009)	30.2 (5.33)	0.021 (0.009)	**0.024**	0.002	0.37
Mid-thigh fat area (cm^2^), significant at <0.05									
BFF (meals/24 h)	22.6 (14.7)	7.33 (1.78)	46 (10.4)	7.33 (1.78)	42.5 (8.6)	7.33 (1.78)	**<0.001**	0.002	0.29

^a^ Data are parameter estimate ± SE (standard error of estimate); effects of predictors taken from linear mixed-effects models that accounted for infant age and interaction between infant age and predictor with a random effect for participant; if the *p*-value for interaction is not <0.05 parameter estimates are taken from a model with no interaction. ^b^ Results are presented only for interactions or predictors with raw *p*-values <0.05. ^c^ Significance for the tested combinations after the FDR adjustment (significant *p*-values are indicated by the bold text). BFF, breastfeeding frequency; MI, milk intake. ^d^ 24-h MI and BFF (meals/24-h) were measured between 2–5 months and within 2 weeks of 9 and 12 months.

## Data Availability

The data presented in this study are available from the corresponding author upon reasonable request.

## References

[1] Leunissen R.W., Kerkhof G.F., Stijnen T., Hok-ken-Koelega A. (2009). Timing and tempo of first-year rapid growth in relation to cardiovascular and metabolic risk profile in early adulthood. JAMA.

[2] Butte N., Wong W., Hopkinson J., Smith E., Ellis K. (2000). Infant feeding mode affects early growth and body composition. Pediatrics.

[3] Woo J.G., Martin J.M. (2015). Does breastfeeding protect against childhood obesity? Moving beyond observational evidence. Curr. Obes. Rep..

[4] De Lucia Rolfe E., Modi N., Uthaya S., Hughes I.A., Dunger D.B., Acerini C., Stolk R.P., Ong K.K. (2013). Ultrasound estimates of visceral and subcutaneous-abdominal adipose tissues in infancy. J. Obes..

[5] Gridneva Z., Rea A., Lai C.T., Tie W.J., Kugananthan S., Murray K., Hartmann P.E., Geddes D.T. (2021). Development of visceral and subcutaneous-abdominal adipose tissue in breastfed infants during first year of lactation. Nutrients.

[6] Golan R., Shelef I., Rudich A., Gepner Y., Shemesh E., Chassidim Y., Harman-Boehm I., Henkin Y., Schwarzfuchs D., Ben Avraham S. (2012). Abdominal superficial subcutaneous fat: A putative distinct protective fat subdepot in type 2 diabetes. Diabetes Care.

[7] Neville M.C., Allen J.C., Archer P.C., Casey C.E., Seacat J., Keller R.P., Lutes V., Rasbach J., Neifert M. (1991). Studies in human lactation: Milk volume and nutrient composition during weaning and lactogenesis. Am. J. Clin. Nutr..

[8] Gridneva Z., George A.D., Suwaydi M.A., Sindi A.S., Ma J., Stinson L.F., Geddes D.T. (2022). Environmental determinants of human milk composition in relation to health outcomes. Acta Paediatr..

[9] Vanderwall C., Clark R.R., Eickhoff J., Carrel A.L. (2017). BMI is a poor predictor of adiposity in young overweight and obese children. BMC Pediatr..

[10] Toro-Ramos T., Paley C., Pi-Sunyer F.X., Gallagher D. (2015). Body composition during fetal development and infancy through the age of 5 years. Eur. J. Clin. Nutr..

[11] Ward L., Poston L., Godfrey K., Koletzko B. (2013). Assessing early growth and adiposity: Report from an Early Nutrition Academy workshop. Ann. Nutr. Metab..

[12] McLeod G., Geddes D., Nathan E., Sherriff J., Simmer K., Hartmann P. (2013). Feasibility of using ultrasound to measure preterm body composition and to assess macronutrient influences on tissue accretion rates. Early Hum. Dev..

[13] Arthur P., Hartmann P., Smith M. (1987). Measurement of the milk intake of breast-fed infants. J. Pediatr. Gastroenterol. Nutr..

[14] Kent J.C., Mitoulas L.R., Cregan M.D., Ramsay D.T., Doherty D.A., Hartmann P.E. (2006). Volume and frequency of breastfeedings and fat content of breast milk throughout the day. Pediatrics.

[15] Larciprete G., Valensise H., Pierro G., Vasapollo B., Casalino B., Arduini D., Jarvis S., Cirese E. (2005). Intrauterine growth restriction and fetal body composition. Ultrasound Obstet. Gynecol..

[16] Gridneva Z., Rea A., Hepworth A.R., Ward L.C., Lai C.T., Hartmann P.E., Geddes D.T. (2018). Relationships between breastfeeding patterns and maternal and infant body composition over the first 12 months of lactation. Nutrients.

[17] Kugananthan S., Gridneva Z., Lai C.T., Hepworth A.R., Mark P.J., Kakulas F., Geddes D.T. (2017). Associations between maternal body composition and appetite hormones and macronutrients in human milk. Nutrients.

[18] Van Itallie T.B., Yang M.U., Heymsfield S.B., Funk R.C., Boileau R.A. (1990). Height-normalized indices of the body’s fat-free mass and fat mass: Potentially useful indicators of nutritional status. Am. J. Clin. Nutr..

[19] Kugananthan S., Lai C.T., Gridneva Z., Mark P.J., Geddes D.T., Kakulas F. (2016). Leptin levels are higher in whole compared to skim human milk, supporting a cellular contribution. Nutrients.

[20] Gridneva Z., Kugananthan S., Rea A., Lai C.T., Ward L.C., Murray K., Hartmann P.E., Geddes D.T. (2018). Human milk adiponectin and leptin and infant body composition over the first 12 months of lactation. Nutrients.

[21] Gridneva Z., Tie W.J., Rea A., Lai C.T., Ward L.C., Murray K., Hartmann P.E., Geddes D.T. (2018). Human milk casein and whey protein and infant body composition over the first 12 months of lactation. Nutrients.

[22] Gridneva Z., Rea A., Tie W.J., Lai C.T., Kugananthan S., Ward L.C., Murray K., Hartmann P.E., Geddes D.T. (2019). Carbohydrates in human milk and body composition of term infants during the first 12 months of lactation. Nutrients.

[23] Gridneva Z., Lai C.T., Rea A., Tie W.J., Ward L.C., Murray K., Hartmann P.E., Geddes D.T. (2020). Human milk immunomodulatory proteins are related to development of infant body composition during the first year of lactation. Pediatr. Res..

[24] Keller R., Neville M. (1986). Determination of total protein in human milk: Comparison of methods. Clin. Chem..

[25] Kunz C., Lonnerdal B. (1989). Human milk proteins: Separation of whey proteins and their analysis by polyacrylamide gel electrophoresis, fast protein liquid chromatography (FPLC) gel filtration, and anion-exchange chromatography. Am. J. Clin. Nutr..

[26] Khan S., Casadio Y., Lai C., Prime D., Hepworth A., Trengove N., Hartmann P. (2012). Investigation of short-term variations in casein and whey proteins in breast milk of term mothers. Hepatol. Nutr..

[27] Mitoulas L.R., Kent J.C., Cox D.B., Owens R.A., Sherriff J.L., Hartmann P.E. (2002). Variation in fat, lactose and protein in human milk over 24 h and throughout the first year of lactation. Br. J. Nutr..

[28] Euber J., Brunner J. (1979). Determination of lactose in milk products by high-performance liquid chromatography. J. Dairy Sci..

[29] Albalasmeh A., Berhe A., Ghezzehei T. (2013). A new method for rapid determination of carbohydrate and total carbon concentrations using UV spectrophotometry. Carbohydr. Polym..

[30] Selsted M., Martinez R. (1980). A simple and ultrasensitive enzymatic assay for the quantitative determination of lysozyme in the picogram range. Anal. Biochem..

[31] Zhang G., Lai C.T., Hartmann P., Oddy W.H., Kusel M.M.H., Sly P.D., Holt P.G. (2014). Anti-infective proteins in breast milk and asthma-associated phenotypes during early childhood. Pediatr. Allergy Immunol..

[32] Tijssen P., Burdon R.H., van Knippenberg P.H. (1985). Practice and theory of immunoessays. Laboratory Techniques in Biochemistry and Molecular Biology.

[33] Diggle P.J., Heagerty P.J., Liang K.-Y., Zeger S.L. (2002). Analysis of Longitudinal Data.

[34] Faul F., Erdfelder E., Buchner A., Lang A.-G. (2009). Statistical power analyses using G*Power 3.1: Tests for correlation and regression analyses. Behav. Res. Methods.

[35] Curran-Everett D. (2000). Multiple comparisons: Philosophies and illustrations. Am. J. Physiol. Regul. Integr. Comp. Physiol..

[36] Kuzawa C.W. (1998). Adipose tissue in human infancy and childhood: An evolutionary perspective. Am. J. Phys. Anthr..

[37] Fomon S., Haschke F., Ziegler E., Nelson S. (1982). Body composition of reference children from birth to age 10 years. Am. J. Clin. Nutr..

[38] Cunnane S.C., Crawford M.A. (2003). Survival of the fattest: Fat babies were the key to evolution of the large human brain. Comp. Biochem. Physiol. Part A Mol. Integr. Physiol..

[39] Adam P.A.J., Raiha N., Rahiala E.-L., Kekomaki M. (1975). Oxidation of glucose and d-β-hydroxybuyrate by the early human fetal brain. Acta Paediatr. Scand..

[40] Carberry A., Golditz P., Lingwood B. (2010). Body composition from birth to 4.5 months in infants born to non-obese women. Pediatr. Res..

[41] Rodríguez-Cano A.M., Mier-Cabrera J., Allegre-Dávalos A.L., Muñoz-Manrique C., Perichart-Perera O. (2019). Higher fat mass and fat mass accretion during the first six months of life in exclusively breastfed infants. Pediatr. Res..

[42] Breij L.M., Abrahamse-Berkeveld M., Acton D., De Lucia Rolfe E., Ong K.K., Hokken-Koelega A.C.S. (2017). Impact of early infant growth, duration of breastfeeding and maternal factors on total body fat mass and visceral fat at 3 and 6 months of age. Ann. Nutr. Metab..

[43] Singh A.S., Mulder C., Twisk J.W.R., Van Mechelen W., Chinapaw M.J.M. (2008). Tracking of childhood overweight into adulthood: A systematic review of the literature. Obes. Rev..

[44] Flegal K.M., Kit B.K., Orpana H., Graubard B.I. (2013). Association of all-cause mortality with overweight and obesity using standard body mass index categories: A systematic review and meta-analysis. JAMA.

[45] Corvalán C., Gregory C.O., Ramirez-Zea M., Martorell R., Stein A.D. (2007). Size at birth, infant, early and later childhood growth and adult body composition: A prospective study in a stunted population. Int. J. Epidemiol..

[46] Booth A., Magnuson A., Foster M. (2014). Detrimental and protective fat: Body fat distribution and its relation to metabolic disease. Horm. Mol. Biol. Clinl. Investig..

[47] Snijder M.B., Visser M., Dekker J.M., Goodpaster B.H., Harris T.B., Kritchevsky S.B., De Rekeneire N., Kanaya A.M., Newman A.B., Tylavsky F.A. (2005). Low subcutaneous thigh fat is a risk factor for unfavourable glucose and lipid levels, independently of high abdominal fat. The Health ABC Study. Diabetologia.

[48] Han E., Lee Y.H., Lee B.W., Kang E.S., Lee I.K., Cha B.S. (2017). Anatomic fat depots and cardiovascular risk: A focus on the leg fat using nationwide surveys (KNHANES 2008–2011). Cardiovasc. Diabetol..

[49] Zhang X., Hu E.A., Wu H., Malik V., Sun Q. (2013). Associations of leg fat accumulation with adiposity-related biological factors and risk of metabolic syndrome. Obesity.

[50] Snijder M.B., Dekker J.M., Visser M., Bouter L.M., Stehouwer C.D., Yudkin J.S., Heine R.J., Nijpels G., Seidell J.C., Hoorn Study (2004). Trunk fat and leg fat have independent and opposite associations with fasting and postload glucose levels: The Hoorn study. Diabetes Care.

[51] Larsson M., Lind M.V., Lharnkjær A., Due A.P., Blom I.C., Wells J., Lai C.T., Mølgaard C., Geddes D.T., Michaelsen K.F. (2018). Excessive weight gain followed by catch-down in exclusively breastfed infants: An exploratory study. Nutrients.

[52] Perrella S.L., Geddes D.T. (2016). A case report of a breastfed infant’s excessive weight gains over 14 months. J. Hum. Lact..

[53] Artym J., Zimecki M. (2013). Milk-derived proteins and peptides in clinical trials. Postepy. Hig. Med. Dosw..

[54] Wang G., Lo L., Forsberg L., Maier R. (2012). Helicobacter pylori peptidoglycan modifications confer lysozyme resistance and contribute to survival in the host. mBio.

[55] Montagne P., Cuilliere M.L., Mole C., Bene M.C., Faure G. (2001). Changes in lactoferrin and lysozyme levels in human milk during the first twelve weeks of lactation. Adv. Exp. Med. Biol..

[56] Bode L. (2015). The functional biology of human milk oligosaccharides. Early Hum. Dev..

[57] Alderete T.A., Autran C., Brekke B.E., Knight R., Bode L., Goran M., Fields D. (2015). Associations between human milk oligosaccharides and infant body composition in the first 6 mo of life. Am. J. Clin. Nutr..

[58] Koleva P.T., Bridgman S.L., Kozyrskyj A.L. (2015). The infant gut microbiome: Evidence for obesity risk and dietary intervention. Nutrients.

[59] Bode L. (2012). Human milk oligosaccharides: Every baby needs a sugar mama. Glycobiology.

[60] Braun O.H., Sandkuhler H. (1985). Relationships between lysozyme concentration of human milk, bacteriologic content, and weight gain of premature infants. J. Pediatr. Gastroenterol. Nutr..

[61] Bol’shakova A.M., Shcherbakova E.G., Ivanova S.D., Medvedeva M.M., Zhuravleva T.P. (1984). Lysozyme in the feeding of premature infants with mixed pathology. Antibiotiki.

[62] Saben J., Sims C., Abraham A., Bode L., Andres A. (2021). Human milk oligosaccharide concentrations and infant intakes are associated with maternal overweight and obesity and predict infant growth. Nutrients.

[63] Woo J., Guerrero M., Altaye M., Ruiz-Palacios G., Martin L., Dubert-Ferrandon A., Newburg D., Morrow A. (2009). Human milk adiponectin is associated with growth in two independent cohorts. Breastfeed Med..

[64] Wang Y.Y., Zhang Z.J., Yao W., Morrow A., Peng Y.M. (2011). Variation of maternal milk adiponectin and its correlation with infant growth. Zhonghua Er Ke Za Zhi.

[65] de Fluiter K., Kerkhof G.F., van Beijsterveldt I., Breij L.M., de Heijning B., Abrahamse-Berkeveld M., Hokken-Koelega A. (2021). Longitudinal human milk macronutrients, body composition and infant appetite during early life. Clin. Nutr..

[66] Dewey K.G., Heinig M.J., Nommsen L.A., Lonnerdal B. (1991). Maternal versus infant factors related to breast milk intake and residual milk volume: The DARLING study. Pediatrics.

[67] Gridneva Z., Kugananthan S., Hepworth A.R., Tie W.J., Lai C.T., Ward L.C., Hartmann P.E., Geddes D.T. (2017). Effect of human milk appetite hormones, macronutrients, and infant characteristics on gastric emptying and breastfeeding patterns of term fully breastfed infants. Nutrients.

[68] Galpin L., Thakwalakwa C., Phuka J., Ashorn P., Maleta K., Wong W.W., Manary M.J. (2007). Breast milk intake is not reduced more by the introduction of energy dense complementary food than by typical infant porridge. J. Nutr..

[69] Cheema A.S., Stinson L.F., Rea A., Lai C.T., Payne M.S., Murray K., Geddes D.T., Gridneva Z. (2021). Human milk lactose, insulin, and glucose relative to infant body composition during exclusive breastfeeding. Nutrients.

[70] Tahir M.J., Haapala J.L., Foster L.P., Duncan K.M., Teague A.M., Kharbanda E.O., McGovern P.M., Whitaker K.M., Rasmussen K.M., Fields D.A. (2019). Higher maternal diet quality during pregnancy and lactation is associated with lower infant weight-for-length, body fat percent, and fat mass in early postnatal life. Nutrients.

[71] Leghi G.E., Netting M.J., Middleton P.F., Wlodek M.E., Geddes D.T., Muhlhausler B.S. (2020). The impact of maternal obesity on human milk macronutrient composition: A systematic review. Nutrients.

[72] Leghi G.E., Netting M.J., Lai C.T., Narayanan A., Dymock M., Rea A., Wlodek M.E., Geddes D.T., Muhlhausler B.S. (2021). Reduction in maternal energy intake during lactation decreased maternal body weight and concentrations of leptin, insulin and adiponectin in human milk without affecting milk production, milk macronutrient composition or infant growth. Nutrients.

